# Supplementary material for: A genetically encoded BRET-based SARS-CoV-2 Mpro protease activity sensor
Source: Commun Chem. 2022 Sep 28;5:117. doi: 10.1038/s42004-022-00731-2 (PMC9516532; doi:10.1038/s42004-022-00731-2)
Supplement: Supplementary file 2 — Supplementary Information [file 42004_2022_731_MOESM2_ESM.pdf]

Supplementary Information

A Genetically encoded BRET-based SARS-CoV-2 M^{pro} protease activity sensor

Anupriya M Geethakumari[1], Wesam S Ahmed[1], Saad Rasool[2], Asma Fatima[1], S.M. Nasir Uddin[1], Mustapha Aouida[1], Kabir H Biswas[1,*]

Affiliations:

[1]Division of Biological and Biomedical Sciences, College of Health & Life Sciences, Hamad Bin Khalifa University, Education City, Qatar Foundation, Doha – 34110, Qatar

[2]Division of Genomics and Precision Medicine, College of Health & Life Sciences, Hamad Bin Khalifa University, Education City, Qatar Foundation, Doha – 34110, Qatar

*Correspondence: kbiswas@hbku.edu.qa

Supplementary Text

BRET-based M^{pro} sensor construct details:

mNG-M^{pro}-Nter-auto-NLuc:

Protein Sequence:

```
MGSSHHHHHHSSGLVPRGSHMVSKGEEDNMASLPATHELHIFGSINGVDFDMVGQGTGNPNDGYEELNLKSTKGDLQ
FSPWILVPHIGYGFHQYLPYPDGMSPFQAAMVDGSGYQVHRTMQFEDGASLTVNYRYTYEGSHIKGEAQVKGTGFPA
DGPVMTNSLTAADWCRSKKTYPNDKTIISTFKWSYTTGNGKRYRSTARTTYTFAKPMAANYLKNQPMYVFRKTELKH
SKTELNFKEWQKAFTDVMGMDELYKEFGTENLYAVLQSGFRGSGGSMVFTLEDFVGDWRQTAGYNLDQVLEQGGVSS
LFQNLGVSVTPIQRIVLSGENGLKIDIHVIIPYEGLSGDQMGQIEKIFKVVYPVDDHHFKVILHYGTLVIDGVTPNM
IDYFGRPYEGIAVFDGKKITVTGTLWNGNKIIDERLINPDGSLLFRVTINGVTGWRLCERILA*
```

Key:

Yellow: His-tag
Green: mNeonGreen
Grey: Linkers
Red: cleavage sequence
Cyan: NLuc

Predicted pI: 6.31
Predicted Mol. Wt.: 50048.54 (~50 kDa)
Predicted Mol. Wt. after cleavage (His-tagged fragment): Theoretical pI/Mw: 6.92 / 30178.78 (30 kDa)

Plasmid DNA Sequence:

```
GACATTGATTATTGACTAGTTATTAATAGTAATCAATTACGGGGTCATTAGTTCATAGCCCATATATGGAGTTCCGCGTTACATAACTTACGGTAAA
TGGCCCGCCTGGCTGACCGCCCAACGACCCCCGCCCATTGACGTCAATAATGACGTATGTTCCCATAGTAACGCCAATAGGGACTTTCCATTGACGT
CAATGGGTGGAGTATTTACGGTAAACTGCCCACTTGGCAGTACATCAAGTGTATCATATGCCAAGTACGCCCCCTATTGACGTCAATGACGGTAAAT
GGCCCGCCTGGCATTATGCCCAGTACATGACCTTATGGGACTTTCCTACTTGGCAGTACATCTACGTATTAGTCATCGCTATTACCATGGTGATGCG
GTTTTGGCAGTACATCAATGGGCGTGGATAGCGGTTTGACTCACGGGGATTTCCAAGTCTCCACCCCATTGACGTCAATGGGAGTTTGTTTTGGCAC
CAAAATCAACGGGACTTTCCAAAATGTCGTAACAACTCCGCCCCATTGACGCAAATGGGCGGTAGGCGTGTACGGTGGGAGGTCTATATAAGCAGAG
CTCTCTGGCTAACTAGAGAACCCACTGCTTACTGGCTTATCGAAATTAATACGACTCACTATAGGGAGACCCAAGCTGGCTAGCGTTTAAACTTAAG
CTTGCCACCATGGGAAGTTCACATCATCATCATCATCACTCATCAGGACTGGTGCCACGGGGGTCTCACATGGTCTCCAAGGGGGAAGAGGACAACA
TGGCCTCTCTGCCTGCCACACACGAGCTGCACATCTTCGGCAGCATCAATGGCGTGGACTTTGATATGGTGGGCCAGGGCACAGGCAACCCAAATGA
CGGCTACGAGGAGCTGAACCTGAAGAGCACCAAGGGCGATCTGCAGTTCTCCCCTTGGATCCTGGTGCCACACATCGGCTATGGCTTTCACCAGTAT
CTGCCCTACCCTGACGGCATGTCCCCCTTCCAGGCCGCCATGGTGGATGGCTCTGGCTACCAGGTGCACAGGACCATGCAGTTTGAGGACGGCGCCT
CCCTGACAGTGAATTACCGGTATACCTACGAGGGCTCTCACATCAAGGGCGAGGCCCAGGTGAAGGGCACAGGCTTCCCAGCCGATGGCCCCGTGAT
GACAAACTCTCTGACCGCCGCCGACTGGTGCCGGAGCAAGAAGACCTACCCTAATGATAAGACAATCATCTCCACCTTTAAGTGGTCTTATACCACA
GGCAACGGCAAGCGGTACAGAAGCACAGCCAGAACCACATATACCTTTGCCAAGCCAATGGCCGCCAACTATCTGAAGAATCAGCCCATGTACGTGT
TCAGGAAGACAGAGCTGAAGCACTCCAAGACCGAGCTGAATTTCAAGGAGTGGCAGAAGGCCTTTACAGACGTGATGGGCATGGATGAGCTGTACAA
GGAGTTTGGCACCGAGAACCTGTATGCAGTGCTCCAAAGCGGATTTCGCGGCTCTGGCGGCAGCATGGTGTTCACACTGGAGGACTTTGTGGGCGAT
TGGCGGCAGACCGCCGGCTATAATCTGGATCAGGTGCTGGAGCAGGGCGGCGTGAGCTCCCTGTTCCAGAACCTGGGCGTGAGCGTGACCCCTATCC
```

AGCGGATCGTGCTGTCCGGCGAGAACGGCCTGAAGATCGACATCCACGTGATCATCCCATACGAGGGCCTGTCTGGCGATCAGATGGGCCAGATCGA
GAAGATCTTCAAGGTGGTGTACCCCGTGGACGATCACCACTTCAAAGTGATCCTGCACTATGGCACCCTGGTCATCGACGGCGTGACCCCCAATATG
ATCGATTATTTCGGCCGGCCTTACGAGGGCATCGCCGTGTTTGACGGCAAGAAGATCACCGTGACAGGCACCCTGTGGAACGGCAATAAGATCATCG
ACGAGAGACTGATCAACCCCGATGGCTCCCTGCTGTTCAGGGTGACTATTAACGGAGTGACTGGCTGGCGGCTGTGCGAAAGGATTCTGGCTTGACT
CGAGTCTAGAGGGCCCGTTTAAACCCGCTGATCAGCCTCGACTGTGCCTTCTAGTTGCCAGCCATCTGTTGTTTGCCCCTCCCCCGTGCCTTCCTTG
ACCCTGGAAGGTGCCACTCCCACTGTCCTTTCCTAATAAAATGAGGAAATTGCATCGCATTGTCTGAGTAGGTGTCATTCTATTCTGGGGGGTGGGG
TGGGGCAGGACAGCAAGGGGGAGGATTGGGAAGACAATAGCAGGCATGCTGGGGATGCGGTGGGCTCTATGGCTTCTGAGGCGGAAAGAACCAGCTG
GGGCTCTAGGGGGTATCCCCACGCGCCCTGTAGCGGCGCATTAAGCGCGGCGGGTGTGGTGGTTACGCGCAGCGTGACCGCTACACTTGCCAGCGCC
CTAGCGCCCGCTCCTTTCGCTTTCTTCCCTTCCTTTCTCGCCACGTTCGCCGGCTTTCCCCGTCAAGCTCTAAATCGGGGGCTCCCTTTAGGGTTCC
GATTTAGTGCTTTACGGCACCTCGACCCCAAAAAACTTGATTAGGGTGATGGTTCACGTAGTGGGCCATCGCCCTGATAGACGGTTTTTCGCCCTTT
GACGTTGGAGTCCACGTTCTTTAATAGTGGACTCTTGTTCCAAACTGGAACAACACTCAACCCTATCTCGGTCTATTCTTTTGATTTATAAGGGATT
TTGCCGATTTCGGCCTATTGGTTAAAAAATGAGCTGATTTAACAAAAATTTAACGCGAATTAATTCTGTGGAATGTGTGTCAGTTAGGGTGTGGAAA
GTCCCCAGGCTCCCCAGCAGGCAGAAGTATGCAAAGCATGCATCTCAATTAGTCAGCAACCAGGTGTGGAAAGTCCCCAGGCTCCCCAGCAGGCAGA
AGTATGCAAAGCATGCATCTCAATTAGTCAGCAACCATAGTCCCGCCCCTAACTCCGCCCATCCCGCCCCTAACTCCGCCCAGTTCCGCCCATTCTC
CGCCCCATGGCTGACTAATTTTTTTTATTTATGCAGAGGCCGAGGCCGCCTCTGCCTCTGAGCTATTCCAGAAGTAGTGAGGAGGCTTTTTTGGAGG
CCTAGGCTTTTGCAAAAAGCTCCCGGGAGCTTGTATATCCATTTTCGGATCTGATCAAGAGACAGGATGAGGATCGTTTCGCATGATTGAACAAGAT
GGATTGCACGCAGGTTCTCCGGCCGCTTGGGTGGAGAGGCTATTCGGCTATGACTGGGCACAACAGACAATCGGCTGCTCTGATGCCGCCGTGTTCC
GGCTGTCAGCGCAGGGGCGCCCGGTTCTTTTTGTCAAGACCGACCTGTCCGGTGCCCTGAATGAACTGCAGGACGAGGCAGCGCGGCTATCGTGGCT
GGCCACGACGGGCGTTCCTTGCGCAGCTGTGCTCGACGTTGTCACTGAAGCGGGAAGGGACTGGCTGCTATTGGGCGAAGTGCCGGGGCAGGATCTC
CTGTCATCTCACCTTGCTCCTGCCGAGAAAGTATCCATCATGGCTGATGCAATGCGGCGGCTGCATACGCTTGATCCGGCTACCTGCCCATTCGACC
ACCAAGCGAAACATCGCATCGAGCGAGCACGTACTCGGATGGAAGCCGGTCTTGTCGATCAGGATGATCTGGACGAAGAGCATCAGGGGCTCGCGCC
AGCCGAACTGTTCGCCAGGCTCAAGGCGCGCATGCCCGACGGCGAGGATCTCGTCGTGACCCATGGCGATGCCTGCTTGCCGAATATCATGGTGGAA
AATGGCCGCTTTTCTGGATTCATCGACTGTGGCCGGCTGGGTGTGGCGGACCGCTATCAGGACATAGCGTTGGCTACCCGTGATATTGCTGAAGAGC
TTGGCGGCGAATGGGCTGACCGCTTCCTCGTGCTTTACGGTATCGCCGCTCCCGATTCGCAGCGCATCGCCTTCTATCGCCTTCTTGACGAGTTCTT
CTGAGCGGGACTCTGGGGTTCGAAATGACCGACCAAGCGACGCCCAACCTGCCATCACGAGATTTCGATTCCACCGCCGCCTTCTATGAAAGGTTGG
GCTTCGGAATCGTTTTCCGGGACGCCGGCTGGATGATCCTCCAGCGCGGGGATCTCATGCTGGAGTTCTTCGCCCACCCCAACTTGTTTATTGCAGC
TTATAATGGTTACAAATAAAGCAATAGCATCACAAATTTCACAAATAAAGCATTTTTTTCACTGCATTCTAGTTGTGGTTTGTCCAAACTCATCAAT
GTATCTTATCATGTCTGTATACCGTCGACCTCTAGCTAGAGCTTGGCGTAATCATGGTCATAGCTGTTTCCTGTGTGAAATTGTTATCCGCTCACAA
TTCCACACAACATACGAGCCGGAAGCATAAAGTGTAAAGCCTGGGGTGCCTAATGAGTGAGCTAACTCACATTAATTGCGTTGCGCTCACTGCCCGC
TTTCCAGTCGGGAAACCTGTCGTGCCAGCTGCATTAATGAATCGGCCAACGCGCGGGGAGAGGCGGTTTGCGTATTGGGCGCTCTTCCGCTTCCTCG
CTCACTGACTCGCTGCGCTCGGTCGTTCGGCTGCGGCGAGCGGTATCAGCTCACTCAAAGGCGGTAATACGGTTATCCACAGAATCAGGGGATAACG
CAGGAAAGAACATGTGAGCAAAAGGCCAGCAAAAGGCCAGGAACCGTAAAAAGGCCGCGTTGCTGGCGTTTTTCCATAGGCTCCGCCCCCCTGACGA
GCATCACAAAAATCGACGCTCAAGTCAGAGGTGGCGAAACCCGACAGGACTATAAAGATACCAGGCGTTTCCCCCTGGAAGCTCCCTCGTGCGCTCT
CCTGTTCCGACCCTGCCGCTTACCGGATACCTGTCCGCCTTTCTCCCTTCGGGAAGCGTGGCGCTTTCTCATAGCTCACGCTGTAGGTATCTCAGTT
CGGTGTAGGTCGTTCGCTCCAAGCTGGGCTGTGTGCACGAACCCCCCGTTCAGCCCGACCGCTGCGCCTTATCCGGTAACTATCGTCTTGAGTCCAA
CCCGGTAAGACACGACTTATCGCCACTGGCAGCAGCCACTGGTAACAGGATTAGCAGAGCGAGGTATGTAGGCGGTGCTACAGAGTTCTTGAAGTGG
TGGCCTAACTACGGCTACACTAGAAGAACAGTATTTGGTATCTGCGCTCTGCTGAAGCCAGTTACCTTCGGAAAAAGAGTTGGTAGCTCTTGATCCG
GCAAACAAACCACCGCTGGTAGCGGTGGTTTTTTTGTTTGCAAGCAGCAGATTACGCGCAGAAAAAAAGGATCTCAAGAAGATCCTTTGATCTTTTC
TACGGGGTCTGACGCTCAGTGGAACGAAAACTCACGTTAAGGGATTTTGGTCATGAGATTATCAAAAAGGATCTTCACCTAGATCCTTTTAAATTAA
AAATGAAGTTTTAAATCAATCTAAAGTATATATGAGTAAACTTGGTCTGACAGTTACCAATGCTTAATCAGTGAGGCACCTATCTCAGCGATCTGTC
TATTTCGTTCATCCATAGTTGCCTGACTCCCCGTCGTGTAGATAACTACGATACGGGAGGGCTTACCATCTGGCCCCAGTGCTGCAATGATACCGCG
AGACCCACGCTCACCGGCTCCAGATTTATCAGCAATAAACCAGCCAGCCGGAAGGGCCGAGCGCAGAAGTGGTCCTGCAACTTTATCCGCCTCCATC
CAGTCTATTAATTGTTGCCGGGAAGCTAGAGTAAGTAGTTCGCCAGTTAATAGTTTGCGCAACGTTGTTGCCATTGCTACAGGCATCGTGGTGTCAC
GCTCGTCGTTTGGTATGGCTTCATTCAGCTCCGGTTCCCAACGATCAAGGCGAGTTACATGATCCCCCATGTTGTGCAAAAAAGCGGTTAGCTCCTT
CGGTCCTCCGATCGTTGTCAGAAGTAAGTTGGCCGCAGTGTTATCACTCATGGTTATGGCAGCACTGCATAATTCTCTTACTGTCATGCCATCCGTA
AGATGCTTTTCTGTGACTGGTGAGTACTCAACCAAGTCATTCTGAGAATAGTGTATGCGGCGACCGAGTTGCTCTTGCCCGGCGTCAATACGGGATA
ATACCGCGCCACATAGCAGAACTTTAAAAGTGCTCATCATTGGAAAACGTTCTTCGGGGCGAAAACTCTCAAGGATCTTACCGCTGTTGAGATCCAG
TTCGATGTAACCCACTCGTGCACCCAACTGATCTTCAGCATCTTTTACTTTCACCAGCGTTTCTGGGTGAGCAAAAACAGGAAGGCAAAATGCCGCA
AAAAAGGGAATAAGGGCGACACGGAAATGTTGAATACTCATACTCTTCCTTTTTCAATATTATTGAAGCATTTATCAGGGTTATTGTCTCATGAGCG
GATACATATTTGAATGTATTTAGAAAAATAAACAAATAGGGGTTCCGCGCACATTTCCCCGAAAAGTGCCACCTGACGTCGACGGATCGGGAGATCT
CCCGATCCCCTATGGTGCACTCTCAGTACAATCTGCTCTGATGCCGCATAGTTAAGCCAGTATCTGCTCCCTGCTTGTGTGTTGGAGGTCGCTGAGT
AGTGCGCGAGCAAAATTTAAGCTACAACAAGGCAAGGCTTGACCGACAATTGCATGAAGAATCTGCTTAGGGTTAGGCGTTTTGCGCTGCTTCGCGA
TGTACGGGCCAGATATACGCGTT

mNG-M^{pro}-Nter-auto-L-NLuc:

Protein Sequence:

MGSSHHHHHHSSGLVPRGSHMVSKGEEDNMASLPATHELHIFGSINGVDFDMVGQGTGNPNDGYEELNLKSTKGDLQ
FSPWILVPHIGYGFHQYLPYPDGMSPFQAAMVDGSGYQVHRTMQFEDGASLTVNYRYTYEGSHIKGEAQVKGTGFPA
DGPVMTNSLTAADWCRSKKTYPNDKTIISTFKWSYTTGNGKRYRSTARTTYTFAKPMAANYLKNQPMYVFRKTELKH
SKTELNFKEWQKAFTDVMGMDELYKEFGTENLYKTSAVLQSGFRKMEGSGGSMVFTLEDFVGDWRQTAGYNLDQVLE

QGGVSSLFQNLGVSVTPIQRIVLSGENGLKIDIHVIIPYEGLSGDQMGQIEKIFKVVYPVDDHHFKVILHYGTLVID

GVTPNMIDYFGRPYEGIAVFDGKKITVTGTLWNGNKIIDERLINPDGSLLFRVTINGVTGWRLCERILA*

Key:

Yellow: His-tag
Green: mNeonGreen
Grey: Linkers
Red: cleavage sequence
Cyan: NLuc

Predicted pI: 6.41
Predicted Mol. Wt.: 50753.38 (~51 kDa)
Predicted Mol. Wt. after cleavage (His-tagged fragment): Theoretical pI/Mw: 7.26 / 30495.14

Plasmid DNA Sequence:

```
GACATTGATTATTGACTAGTTATTAATAGTAATCAATTACGGGGTCATTAGTTCATAGCCCATATATGGAGTTCCGCGTTACATAACTTACGGTAAA
TGGCCCGCCTGGCTGACCGCCCAACGACCCCCGCCCATTGACGTCAATAATGACGTATGTTCCCATAGTAACGCCAATAGGGACTTTCCATTGACGT
CAATGGGTGGAGTATTTACGGTAAACTGCCCACTTGGCAGTACATCAAGTGTATCATATGCCAAGTACGCCCCCTATTGACGTCAATGACGGTAAAT
GGCCCGCCTGGCATTATGCCCAGTACATGACCTTATGGGACTTTCCTACTTGGCAGTACATCTACGTATTAGTCATCGCTATTACCATGGTGATGCG
GTTTTGGCAGTACATCAATGGGCGTGGATAGCGGTTTGACTCACGGGGATTTCCAAGTCTCCACCCCATTGACGTCAATGGGAGTTTGTTTTGGCAC
CAAAATCAACGGGACTTTCCAAAATGTCGTAACAACTCCGCCCCATTGACGCAAATGGGCGGTAGGCGTGTACGGTGGGAGGTCTATATAAGCAGAG
CTCTCTGGCTAACTAGAGAACCCACTGCTTACTGGCTTATCGAAATTAATACGACTCACTATAGGGAGACCCAAGCTGGCTAGCGTTTAAACTTAAG
CTTGCCACCATGGGAAGTTCACATCATCATCATCATCACTCATCAGGACTGGTGCCACGGGGGTCTCACATGGTCTCCAAGGGGGAAGAGGACAACA
TGGCCTCTCTGCCTGCCACACACGAGCTGCACATCTTCGGCAGCATCAATGGCGTGGACTTTGATATGGTGGGCCAGGGCACAGGCAACCCAAATGA
CGGCTACGAGGAGCTGAACCTGAAGAGCACCAAGGGCGATCTGCAGTTCTCCCCTTGGATCCTGGTGCCACACATCGGCTATGGCTTTCACCAGTAT
CTGCCCTACCCTGACGGCATGTCCCCCTTCCAGGCCGCCATGGTGGATGGCTCTGGCTACCAGGTGCACAGGACCATGCAGTTTGAGGACGGCGCCT
CCCTGACAGTGAATTACCGGTATACCTACGAGGGCTCTCACATCAAGGGCGAGGCCCAGGTGAAGGGCACAGGCTTCCCAGCCGATGGCCCCGTGAT
GACAAACTCTCTGACCGCCGCCGACTGGTGCCGGAGCAAGAAGACCTACCCTAATGATAAGACAATCATCTCCACCTTTAAGTGGTCTTATACCACA
GGCAACGGCAAGCGGTACAGAAGCACAGCCAGAACCACATATACCTTTGCCAAGCCAATGGCCGCCAACTATCTGAAGAATCAGCCCATGTACGTGT
TCAGGAAGACAGAGCTGAAGCACTCCAAGACCGAGCTGAATTTCAAGGAGTGGCAGAAGGCCTTTACAGACGTGATGGGCATGGATGAGCTGTACAA
GGAGTTTGGCACCGAGAACCTGTATAAAACGAGTGCCGTATTGCAGAGTGGGTTTCGGAAAATGGAAGGCTCTGGCGGCAGCATGGTGTTCACACTG
GAGGACTTTGTGGGCGATTGGCGGCAGACCGCCGGCTATAATCTGGATCAGGTGCTGGAGCAGGGCGGCGTGAGCTCCCTGTTCCAGAACCTGGGCG
TGAGCGTGACCCCTATCCAGCGGATCGTGCTGTCCGGCGAGAACGGCCTGAAGATCGACATCCACGTGATCATCCCATACGAGGGCCTGTCTGGCGA
TCAGATGGGCCAGATCGAGAAGATCTTCAAGGTGGTGTACCCCGTGGACGATCACCACTTCAAAGTGATCCTGCACTATGGCACCCTGGTCATCGAC
GGCGTGACCCCCAATATGATCGATTATTTCGGCCGGCCTTACGAGGGCATCGCCGTGTTTGACGGCAAGAAGATCACCGTGACAGGCACCCTGTGGA
ACGGCAATAAGATCATCGACGAGAGACTGATCAACCCCGATGGCTCCCTGCTGTTCAGGGTGACTATTAACGGAGTGACTGGCTGGCGGCTGTGCGA
AAGGATTCTGGCTTGACTCGAGTCTAGAGGGCCCGTTTAAACCCGCTGATCAGCCTCGACTGTGCCTTCTAGTTGCCAGCCATCTGTTGTTTGCCCC
TCCCCCGTGCCTTCCTTGACCCTGGAAGGTGCCACTCCCACTGTCCTTTCCTAATAAAATGAGGAAATTGCATCGCATTGTCTGAGTAGGTGTCATT
CTATTCTGGGGGGTGGGGTGGGGCAGGACAGCAAGGGGGAGGATTGGGAAGACAATAGCAGGCATGCTGGGGATGCGGTGGGCTCTATGGCTTCTGA
GGCGGAAAGAACCAGCTGGGGCTCTAGGGGGTATCCCCACGCGCCCTGTAGCGGCGCATTAAGCGCGGCGGGTGTGGTGGTTACGCGCAGCGTGACC
GCTACACTTGCCAGCGCCCTAGCGCCCGCTCCTTTCGCTTTCTTCCCTTCCTTTCTCGCCACGTTCGCCGGCTTTCCCCGTCAAGCTCTAAATCGGG
GGCTCCCTTTAGGGTTCCGATTTAGTGCTTTACGGCACCTCGACCCCAAAAAACTTGATTAGGGTGATGGTTCACGTAGTGGGCCATCGCCCTGATA
GACGGTTTTTCGCCCTTTGACGTTGGAGTCCACGTTCTTTAATAGTGGACTCTTGTTCCAAACTGGAACAACACTCAACCCTATCTCGGTCTATTCT
TTTGATTTATAAGGGATTTTGCCGATTTCGGCCTATTGGTTAAAAAATGAGCTGATTTAACAAAAATTTAACGCGAATTAATTCTGTGGAATGTGTG
TCAGTTAGGGTGTGGAAAGTCCCCAGGCTCCCCAGCAGGCAGAAGTATGCAAAGCATGCATCTCAATTAGTCAGCAACCAGGTGTGGAAAGTCCCCA
GGCTCCCCAGCAGGCAGAAGTATGCAAAGCATGCATCTCAATTAGTCAGCAACCATAGTCCCGCCCCTAACTCCGCCCATCCCGCCCCTAACTCCGC
CCAGTTCCGCCCATTCTCCGCCCCATGGCTGACTAATTTTTTTTATTTATGCAGAGGCCGAGGCCGCCTCTGCCTCTGAGCTATTCCAGAAGTAGTG
AGGAGGCTTTTTTGGAGGCCTAGGCTTTTGCAAAAAGCTCCCGGGAGCTTGTATATCCATTTTCGGATCTGATCAAGAGACAGGATGAGGATCGTTT
CGCATGATTGAACAAGATGGATTGCACGCAGGTTCTCCGGCCGCTTGGGTGGAGAGGCTATTCGGCTATGACTGGGCACAACAGACAATCGGCTGCT
CTGATGCCGCCGTGTTCCGGCTGTCAGCGCAGGGGCGCCCGGTTCTTTTTGTCAAGACCGACCTGTCCGGTGCCCTGAATGAACTGCAGGACGAGGC
AGCGCGGCTATCGTGGCTGGCCACGACGGGCGTTCCTTGCGCAGCTGTGCTCGACGTTGTCACTGAAGCGGGAAGGGACTGGCTGCTATTGGGCGAA
GTGCCGGGGCAGGATCTCCTGTCATCTCACCTTGCTCCTGCCGAGAAAGTATCCATCATGGCTGATGCAATGCGGCGGCTGCATACGCTTGATCCGG
CTACCTGCCCATTCGACCACCAAGCGAAACATCGCATCGAGCGAGCACGTACTCGGATGGAAGCCGGTCTTGTCGATCAGGATGATCTGGACGAAGA
GCATCAGGGGCTCGCGCCAGCCGAACTGTTCGCCAGGCTCAAGGCGCGCATGCCCGACGGCGAGGATCTCGTCGTGACCCATGGCGATGCCTGCTTG
CCGAATATCATGGTGGAAAATGGCCGCTTTTCTGGATTCATCGACTGTGGCCGGCTGGGTGTGGCGGACCGCTATCAGGACATAGCGTTGGCTACCC
```

```
GTGATATTGCTGAAGAGCTTGGCGGCGAATGGGCTGACCGCTTCCTCGTGCTTTACGGTATCGCCGCTCCCGATTCGCAGCGCATCGCCTTCTATCG
CCTTCTTGACGAGTTCTTCTGAGCGGGACTCTGGGGTTCGAAATGACCGACCAAGCGACGCCCAACCTGCCATCACGAGATTTCGATTCCACCGCCG
CCTTCTATGAAAGGTTGGGCTTCGGAATCGTTTTCCGGGACGCCGGCTGGATGATCCTCCAGCGCGGGGATCTCATGCTGGAGTTCTTCGCCCACCC
CAACTTGTTTATTGCAGCTTATAATGGTTACAAATAAAGCAATAGCATCACAAATTTCACAAATAAAGCATTTTTTTCACTGCATTCTAGTTGTGGT
TTGTCCAAACTCATCAATGTATCTTATCATGTCTGTATACCGTCGACCTCTAGCTAGAGCTTGGCGTAATCATGGTCATAGCTGTTTCCTGTGTGAA
ATTGTTATCCGCTCACAATTCCACACAACATACGAGCCGGAAGCATAAAGTGTAAAGCCTGGGGTGCCTAATGAGTGAGCTAACTCACATTAATTGC
GTTGCGCTCACTGCCCGCTTTCCAGTCGGGAAACCTGTCGTGCCAGCTGCATTAATGAATCGGCCAACGCGCGGGGAGAGGCGGTTTGCGTATTGGG
CGCTCTTCCGCTTCCTCGCTCACTGACTCGCTGCGCTCGGTCGTTCGGCTGCGGCGAGCGGTATCAGCTCACTCAAAGGCGGTAATACGGTTATCCA
CAGAATCAGGGGATAACGCAGGAAAGAACATGTGAGCAAAAGGCCAGCAAAAGGCCAGGAACCGTAAAAAGGCCGCGTTGCTGGCGTTTTTCCATAG
GCTCCGCCCCCCTGACGAGCATCACAAAAATCGACGCTCAAGTCAGAGGTGGCGAAACCCGACAGGACTATAAAGATACCAGGCGTTTCCCCCTGGA
AGCTCCCTCGTGCGCTCTCCTGTTCCGACCCTGCCGCTTACCGGATACCTGTCCGCCTTTCTCCCTTCGGGAAGCGTGGCGCTTTCTCATAGCTCAC
GCTGTAGGTATCTCAGTTCGGTGTAGGTCGTTCGCTCCAAGCTGGGCTGTGTGCACGAACCCCCCGTTCAGCCCGACCGCTGCGCCTTATCCGGTAA
CTATCGTCTTGAGTCCAACCCGGTAAGACACGACTTATCGCCACTGGCAGCAGCCACTGGTAACAGGATTAGCAGAGCGAGGTATGTAGGCGGTGCT
ACAGAGTTCTTGAAGTGGTGGCCTAACTACGGCTACACTAGAAGAACAGTATTTGGTATCTGCGCTCTGCTGAAGCCAGTTACCTTCGGAAAAAGAG
TTGGTAGCTCTTGATCCGGCAAACAAACCACCGCTGGTAGCGGTGGTTTTTTTGTTTGCAAGCAGCAGATTACGCGCAGAAAAAAAGGATCTCAAGA
AGATCCTTTGATCTTTTCTACGGGGTCTGACGCTCAGTGGAACGAAAACTCACGTTAAGGGATTTTGGTCATGAGATTATCAAAAAGGATCTTCACC
TAGATCCTTTTAAATTAAAAATGAAGTTTTAAATCAATCTAAAGTATATATGAGTAAACTTGGTCTGACAGTTACCAATGCTTAATCAGTGAGGCAC
CTATCTCAGCGATCTGTCTATTTCGTTCATCCATAGTTGCCTGACTCCCCGTCGTGTAGATAACTACGATACGGGAGGGCTTACCATCTGGCCCCAG
TGCTGCAATGATACCGCGAGACCCACGCTCACCGGCTCCAGATTTATCAGCAATAAACCAGCCAGCCGGAAGGGCCGAGCGCAGAAGTGGTCCTGCA
ACTTTATCCGCCTCCATCCAGTCTATTAATTGTTGCCGGGAAGCTAGAGTAAGTAGTTCGCCAGTTAATAGTTTGCGCAACGTTGTTGCCATTGCTA
CAGGCATCGTGGTGTCACGCTCGTCGTTTGGTATGGCTTCATTCAGCTCCGGTTCCCAACGATCAAGGCGAGTTACATGATCCCCCATGTTGTGCAA
AAAAGCGGTTAGCTCCTTCGGTCCTCCGATCGTTGTCAGAAGTAAGTTGGCCGCAGTGTTATCACTCATGGTTATGGCAGCACTGCATAATTCTCTT
ACTGTCATGCCATCCGTAAGATGCTTTTCTGTGACTGGTGAGTACTCAACCAAGTCATTCTGAGAATAGTGTATGCGGCGACCGAGTTGCTCTTGCC
CGGCGTCAATACGGGATAATACCGCGCCACATAGCAGAACTTTAAAAGTGCTCATCATTGGAAAACGTTCTTCGGGGCGAAAACTCTCAAGGATCTT
ACCGCTGTTGAGATCCAGTTCGATGTAACCCACTCGTGCACCCAACTGATCTTCAGCATCTTTTACTTTCACCAGCGTTTCTGGGTGAGCAAAAACA
GGAAGGCAAAATGCCGCAAAAAAGGGAATAAGGGCGACACGGAAATGTTGAATACTCATACTCTTCCTTTTTCAATATTATTGAAGCATTTATCAGG
GTTATTGTCTCATGAGCGGATACATATTTGAATGTATTTAGAAAAATAAACAAATAGGGGTTCCGCGCACATTTCCCCGAAAAGTGCCACCTGACGT
CGACGGATCGGGAGATCTCCCGATCCCCTATGGTGCACTCTCAGTACAATCTGCTCTGATGCCGCATAGTTAAGCCAGTATCTGCTCCCTGCTTGTG
TGTTGGAGGTCGCTGAGTAGTGCGCGAGCAAAATTTAAGCTACAACAAGGCAAGGCTTGACCGACAATTGCATGAAGAATCTGCTTAGGGTTAGGCG
TTTTGCGCTGCTTCGCGATGTACGGGCCAGATATACGCGTT
```

M^{pro} protease constructs details:

WT M^{pro} Sequence:

As expressed from the pLVX-EF1alpha-SARS-CoV-2-nsp5-2xStrep-IRES-Puro[1] plasmid

```
MSGFRKMAFPSGKVEGCMVQVTCGTTTLNGLWLDDVVYCPRHVICTSEDMLNPNYEDLLIRKSN
HNFLVQAGNVQLRVIGHSMQNCVLKLKVDTANPKTPKYKFVRIQPGQTFSVLACYNGSPSGVYQ
CAMRPNFTIKGSFLNGSCGSVGFNIDYDCVSFCYMHHMELPTGVHAGTDLEGNFYGPFVDRQTA
QAAGTDTTITVNVLAWLYAAVINGDRWFLNRFTTTLNDFNLVAMKYNYEPLTQDHVDILGPLSA
QTGIAVLDMCASLKELLQNGMNGRTILGSALLEDEFTPFDVVRQCSGVTFQLEGGGGWSHPQFE
KGGGSGGGSGGGSWSHPQFEK
```

C145A mutant M^{pro} Sequence:

As expressed from the pLVX-EF1alpha-SARS-CoV-2-nsp5-C145A-2xStrep-IRES-Puro[1] plasmid

MSGFRKMAFPSGKVEGCMVQVTCGTTTLNGLWLDDVVYCPRHVICTSEDMLNPNYEDLLIRKSN

HNFLVQAGNVQLRVIGHSMQNCVLKLKVDTANPKTPKYKFVRIQPGQTFSVLACYNGSPSGVYQ

CAMRPNFTIKGSFLNGSAGSVGFNIDYDCVSFCYMHHMELPTGVHAGTDLEGNFYGPFVDRQTA

QAAGTDTTITVNVLAWLYAAVINGDRWFLNRFTTTLNDFNLVAMKYNYEPLTQDHVDILGPLSA

QTGIAVLDMCASLKELLQNGMNGRTILGSALLEDEFTPFDVVRQCSGVTFQLEGGGGWSHPQFE

KGGGSGGGSGGGSWSHPQFEK

Key:
Green: M^{pro}
Yellow: Strep-tag II
Red: C145A mutation

Supplementary Methods

Dynamic Light Scattering (DLS) measurements

DLS measurements of recombinantly purified proteins were performed using the Zetasizer Nano ZS (Malvern Panalytical, Malvern, United Kingdom). Proteins, either bovine serum albumin (BSA; Tocris Bioscience, Cat. No. 5217) or the short M^{pro} sensor, were prepared in 1× TBS at a final concentration of 1 µM and 400 nM, respectively and light scattering measurements were performed. The purified short M^{pro} biosensor protein was centrifuged prior to measurement at 14000 rpm for 1 hour at 4 °C and supernatant taken to remove any aggregates. Multiple size spectra (N = 40 for BSA and 61 for the short M^{pro} biosensor) obtained from triplicate measurements for 5 s were used for the determination of average molecular size of the proteins.

Script used for determining FlipGFP-based M^{pro} sensor activity in live cells

The following script was used for determining the percentage of GF positive (GFP^+), number of transfected cells per image frame and total number of cells analyzed for each time points.

```
dir_img = getDirectory("Choose your directory of images");
list_img = getFileList(dir_img);

setBatchMode(true);

for (i=0; i<list_img.length; i++) {

open(dir_img + list_img[i]);

name=getTitle;
run("Split Channels");

selectWindow("C1-" +name);
run("Duplicate...", "title=duplicate");

selectWindow("duplicate");
run("Subtract Background...", "rolling=5");
run("Enhance Contrast...", "saturated=0.3");
```

```
run("Gaussian Blur...", "sigma=1");
run("Auto Local Threshold", "method=Bernsen radius=5 parameter_1=0
parameter_2=0 white");
run("Analyze Particles...", "size=5-Infinity circularity=0.10-1.00
add");

selectWindow("duplicate");
run("Close");

selectWindow("C1-" +name);
roiManager("Measure");
//print("C1-" +name);
selectWindow("C2-" +name);
roiManager("Measure");

      selectWindow("Results");
      saveAs("Text", dir_img+name+".txt");
      run("Close");

roiManager("Delete");

selectWindow("C1-" +name);
run("Close");

selectWindow("C2-" +name);
run("Close");
}
run("Close All");
```

Bacterial expression and purification of M^{pro} sensor

The mNG-M^{pro}-Nter-auto-NLuc (short) sensor was expressed in *Escherichia coli* (*E. coli*) BL21-CodonPlus cells (Agilent Technologies) in 100 mL of LB medium, as described previously[2]. Protein expression was induced by the addition of 0.5 mM isopropyl-β-D-thiogalactopyranoside (IPTG), followed by overnight incubation at 20℃. After harvesting the cells by centrifugation (10000 g, 10 min, 4℃), the pellet was resuspended in lysis buffer (10 mL per gram cell pellet; 10 mM phosphate buffer, 2.7 mM KCl, 507 mM NaCl, 10% glycerol, 20 mM imidazole and 0.1 mM DTT), followed by sonication. The supernatant was collected after centrifugation (18000 g, 90

min, 45°C). The sensor construct was purified using Ni-NTA affinity chromatography. The concentration of the sensor was determined using Bradford assay[3].

In vitro enzyme kinetics measurements

To determine the initial reaction velocity, a range of the short M^{pro} sensor concentrations were incubated with 200 and 500 nM of the recombinantly purified M^{pro} protein in a buffer containing 50 mM HEPES, 50 mM NaCl, 0.1% Triton X-100, 1 mM 1 mM Dithiothreitol (DTT) & 1 mM ethylenediamine tetraacetic acid (EDTA) in a total volume of 50 µL for 2.25 h at 37°C. Following incubation, each reaction was diluted to a final concentration of 20 nM of the M^{pro} sensor and BRET measured using a Tecan SPARK® multimode microplate reader after addition of NLuc substrate. For initial reaction velocity calculation at each sensor (substrate) concentration, a background BRET value of 0.25 (obtained using NLuc alone) was subtracted from the initial as well as final BRET values. Reaction rates were then calculated as:

$$Rate = {[(BRET^{Initial} - BRET^{Final}) \times [Mpro\ sensor]} / {time}$$

where Rate = reaction velocity at a M^{pro} sensor concentration

$BRET^{initial}$ and $BRET^{final}$ = initial and final BRET ratio, respectively,

[M^{pro} sensor] = concentration of M^{pro} sensor

time = incubation time

Supplementary Figures

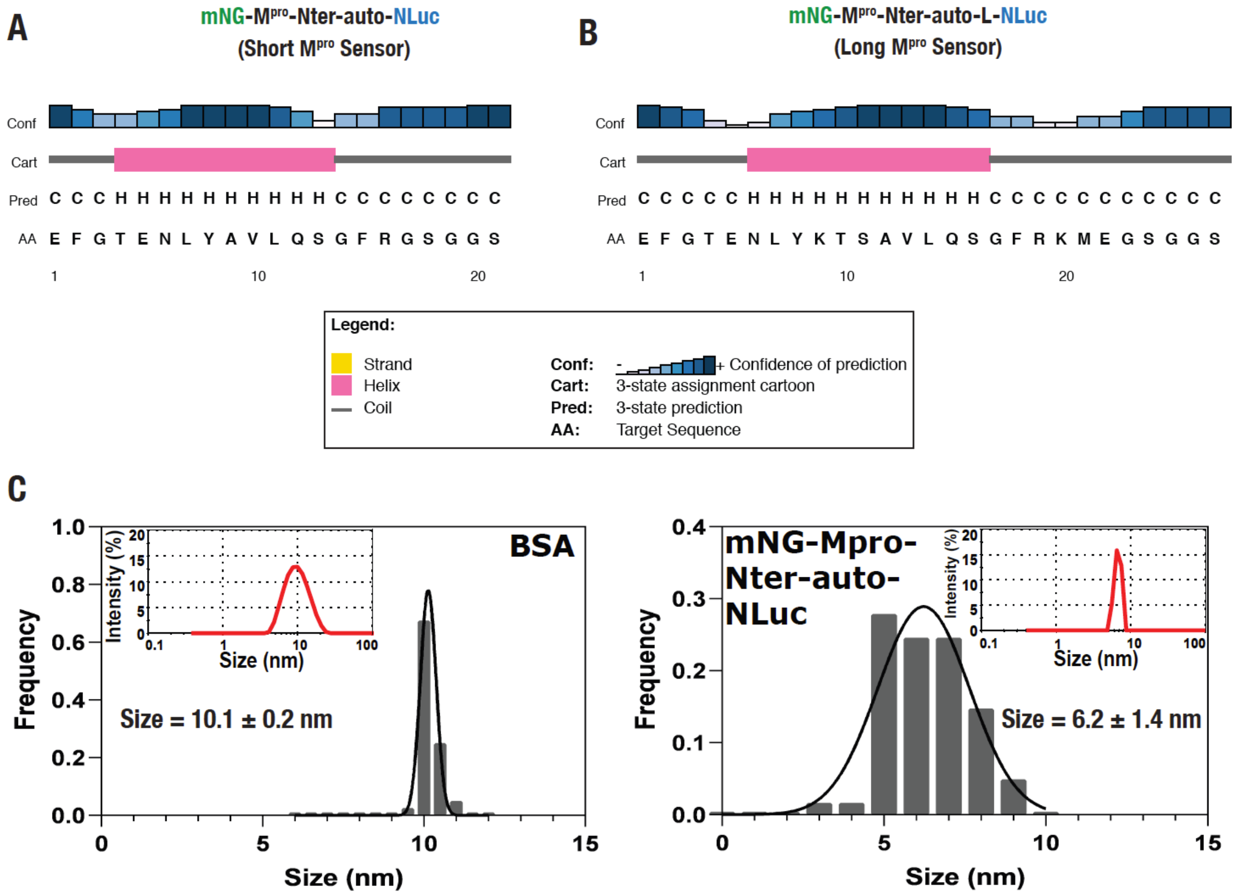

Supplementary Figure 1. Secondary structure prediction of the linkers and Dynamic Light Scattering (DLS) measurements of M^{pro} sensor. (A,B) Secondary structure prediction of the short (A) and long (B) M^{pro} BRET biosensor linkers containing M^{pro} cleavage sites. (C) Graph showing frequency distribution of size (diameter, nm) of BSA (left panel) and the short M^{pro} biosensor (right panel) determined from multiple (N = 40 for BSA and 61 from the short M^{pro} biosensor) DLS measurements. Insets in the respective graphs show a representative measurement.

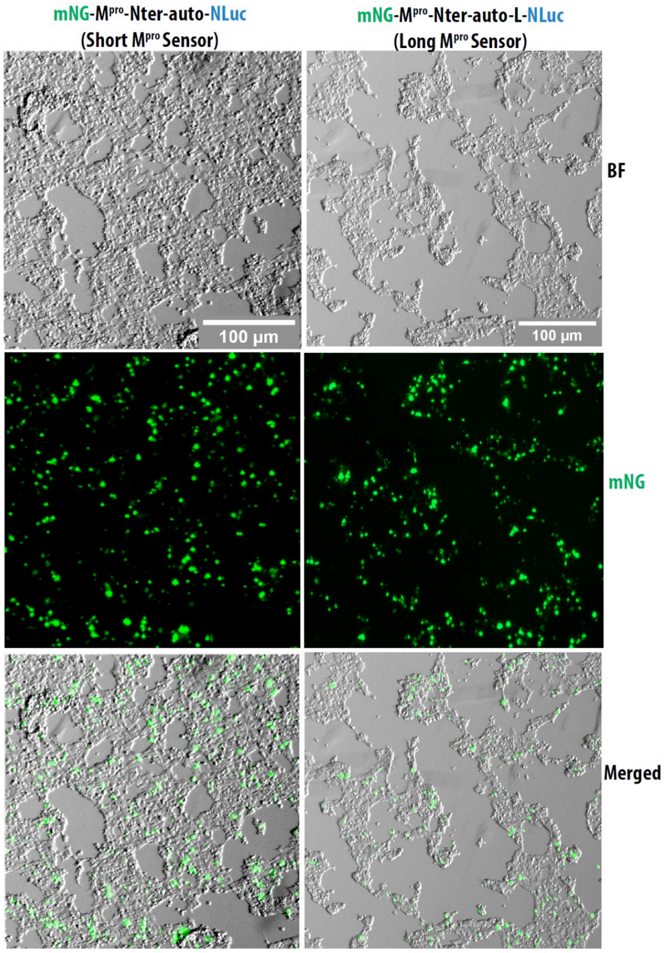

Supplementary Figure 2. Fluorescence image of live cells showing expression of the M^{pro} sensor. Epifluorescence images acquired using a 4× objective of HEK 293T cells transfected with either pmNG-M^{pro}-Nter-auto-NLuc (short; left panel) or pmNG-M^{pro}-Nter-auto-L-NLuc (long; right panel) plasmids showing robust expression of the sensor constructs in these cells.

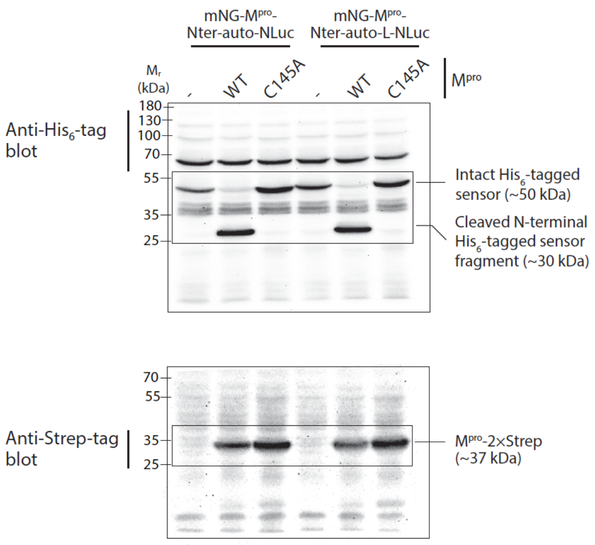

Supplementary Figure 3. Western blot images of the M^{pro} sensor constructs and M^{pro}. Full western blot images used for preparing Fig. 3H top and bottom panels, respectively. Cropped regions used for preparing Fig. 3H are indicated with the rectangles in respective blots.

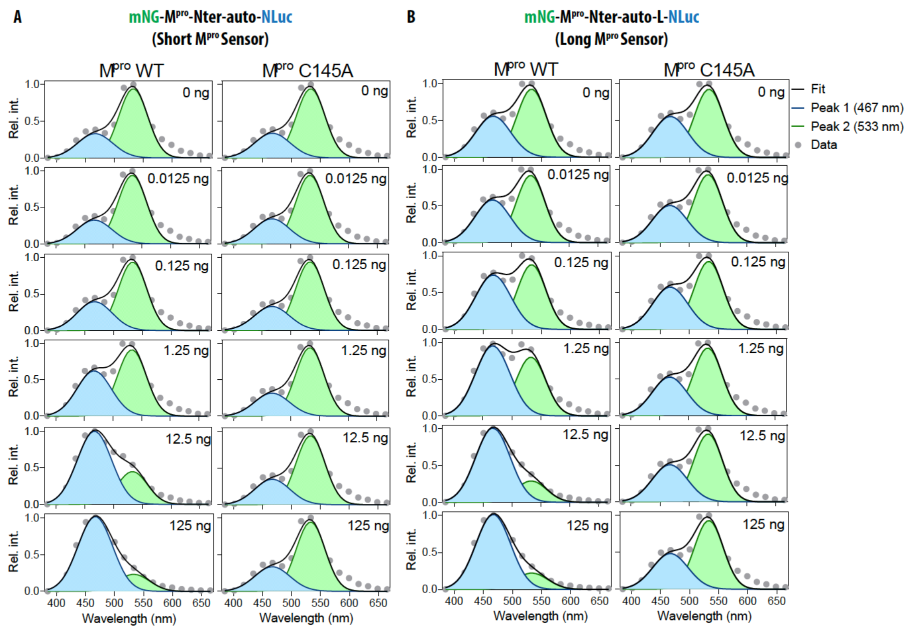

Supplementary Figure 4. M^{pro} plasmid DNA dose-dependent cleavage of the M^{pro} sensors in live cells. (A,B) Graphs showing bioluminescence spectra of the short (A) and long (B) M^{pro} sensor constructs in cells expressing either the WT or C145A mutant M^{pro} protease. Data were fit to a two Gaussian model reflecting mNG fluorescence and NLuc bioluminescence peaks. Note the dose-dependent cleavage of both the short (A) as well as the long (B) sensors specifically in the presence of the WT M^{pro} as reflected by the reduction in the mNG peak (533 nm) of the sensor constructs. Data shown are mean ± S.D. from a representative of independent experiments, with each experiment performed in triplicates.

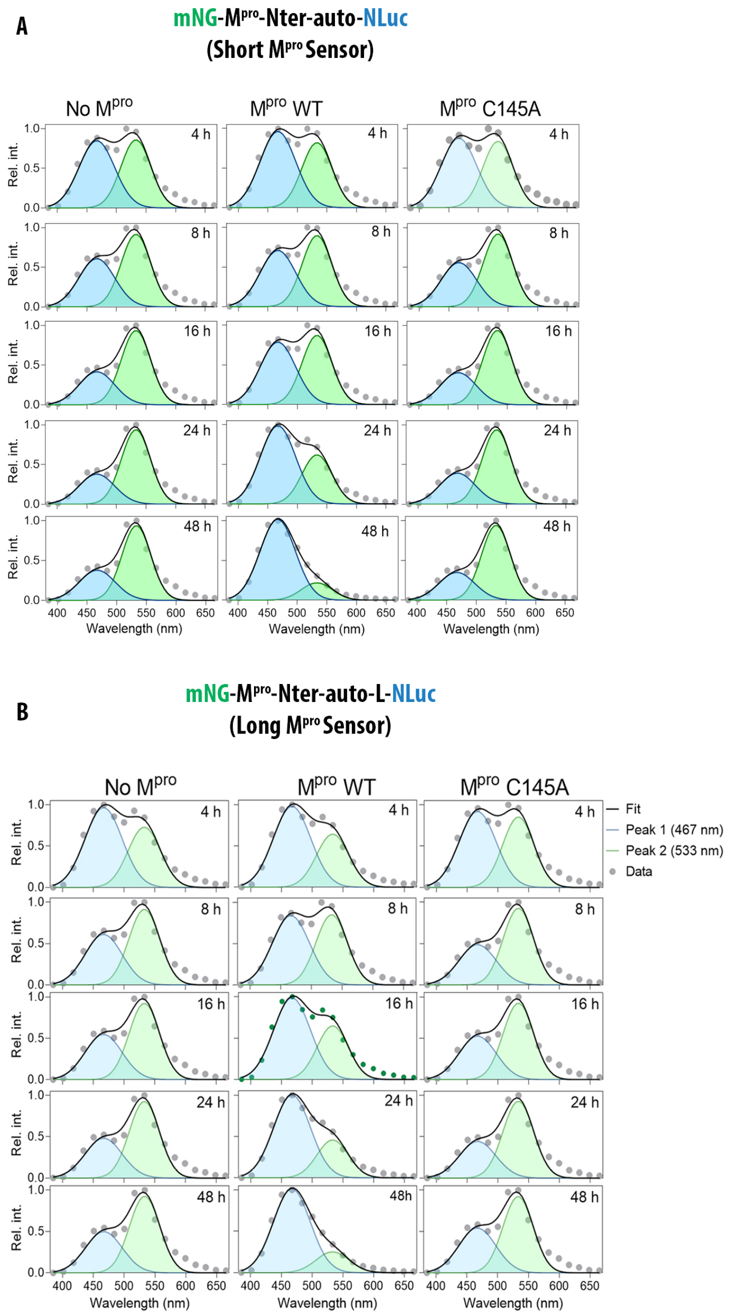

Supplementary Figure 5. Temporal dynamics of M^{pro} protease activity in live cells. (A, B) Graphs showing bioluminescence spectra of the short (A) and long (B) M^{pro} sensor constructs either in control cells or in cells expressing the WT or C145A mutant M^{pro} protease. Data were fit to a two Gaussian model reflecting mNG fluorescence and NLuc bioluminescence peaks. Note the time-dependent cleavage of short (A) and long (B) sensors specifically in the presence

of the WT M^{pro} as reflected by the reduction in the mNG peak (533 nm) of the sensor constructs. Data shown are mean ± S.D. from a representative of independent experiments performed thrice.

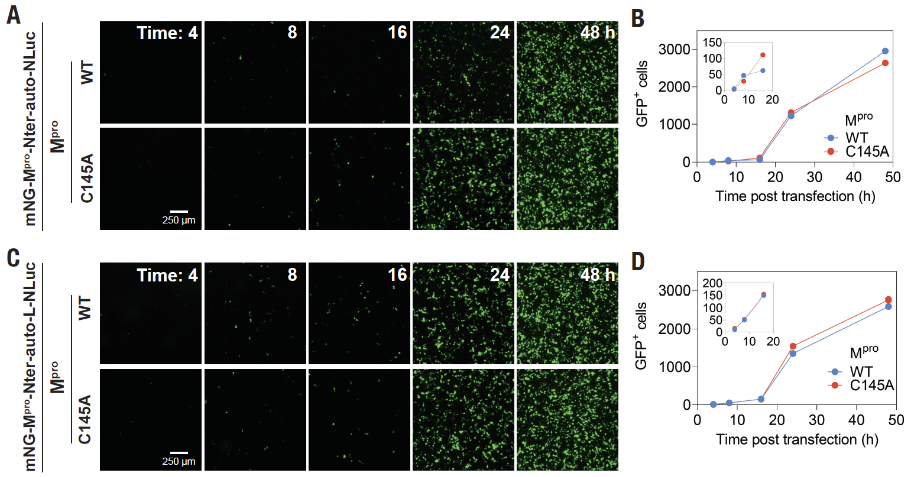

Supplementary Figure 6. Time-dependent expression of the BRET-based M^{pro} sensors. (A,C) Epifluorescence images acquired using a 4× objective of HEK 293T cells transfected with either pmNG-M^{pro}-Nter-auto-NLuc (short; A) or pmNG-M^{pro}-Nter-auto-L-NLuc (long; C) plasmids showing a time-dependent increase in the number of cells expressing the sensors. (B,D) Graph showing time-dependent increase in GFP^{+} cells after transfection with either pmNG-M^{pro}-Nter-auto-NLuc (short; B) or pmNG-M^{pro}-Nter-auto-L-NLuc (long; D) plasmids. Data shown are mean ± S.D. from a representative of independent experiments, with each experiment performed in triplicates.

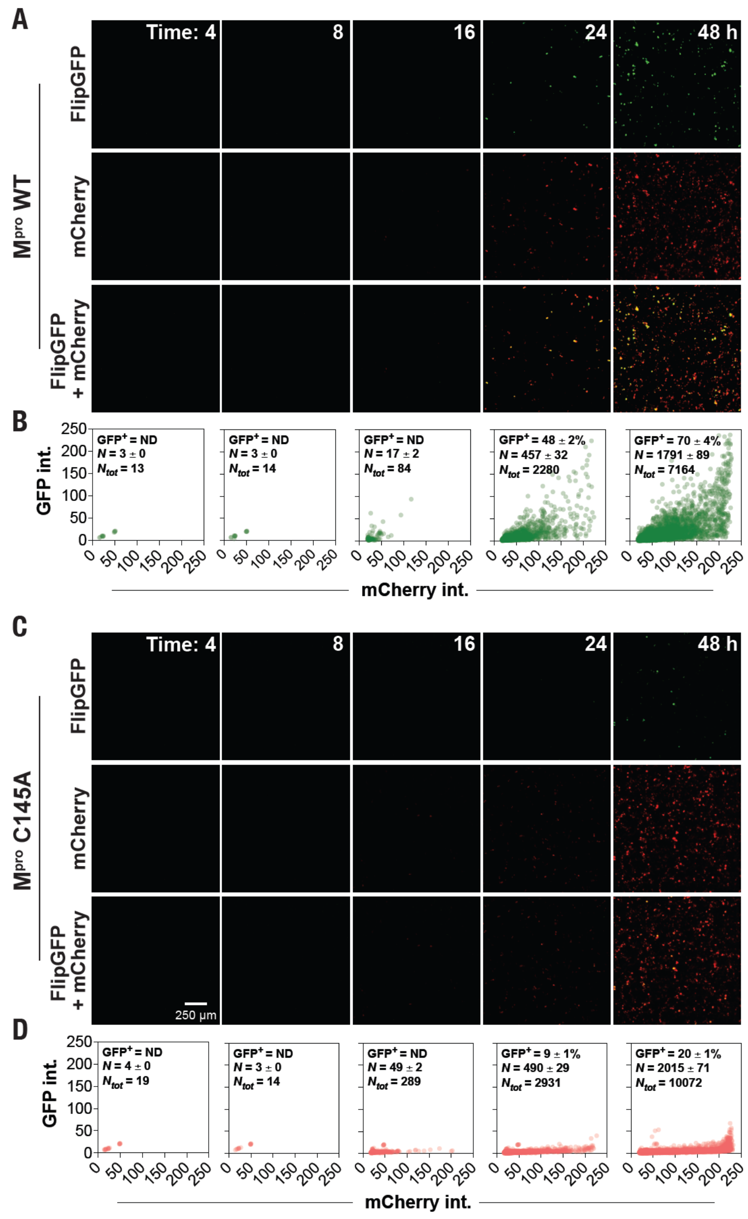

Supplementary Figure 7. Monitoring M^{pro} proteolytic activity using the FlipGFP-based Mpro sensor in live cells. (A) Epifluorescence images of cells showing time-dependent expression of GFP, which is converted from the non-fluorescent FlipGFP upon proteolytic cleavage by M^{pro} (top panel), mCherry (middle panel) and merge (bottom panel) in cells transfected with the WT M^{pro}. (B) Graphs showing GFP and mCherry fluorescence in individual cells transfected with the WT M^{pro} at the indicated time points. (C) Epifluorescence images of

cells showing time-dependent expression of GFP (top panel), mCherry (middle panel) and merge (bottom panel) in cells transfected with the C145A mutant M^{pro}. (D) Graphs showing GFP and mCherry fluorescence in individual cells transfected with the C145A mutant M^{pro} at the indicated time points. (E) Graph showing % GFP^+ cells at the indicated time post transfection with either the wild type or the C145A mutant M^{pro}.

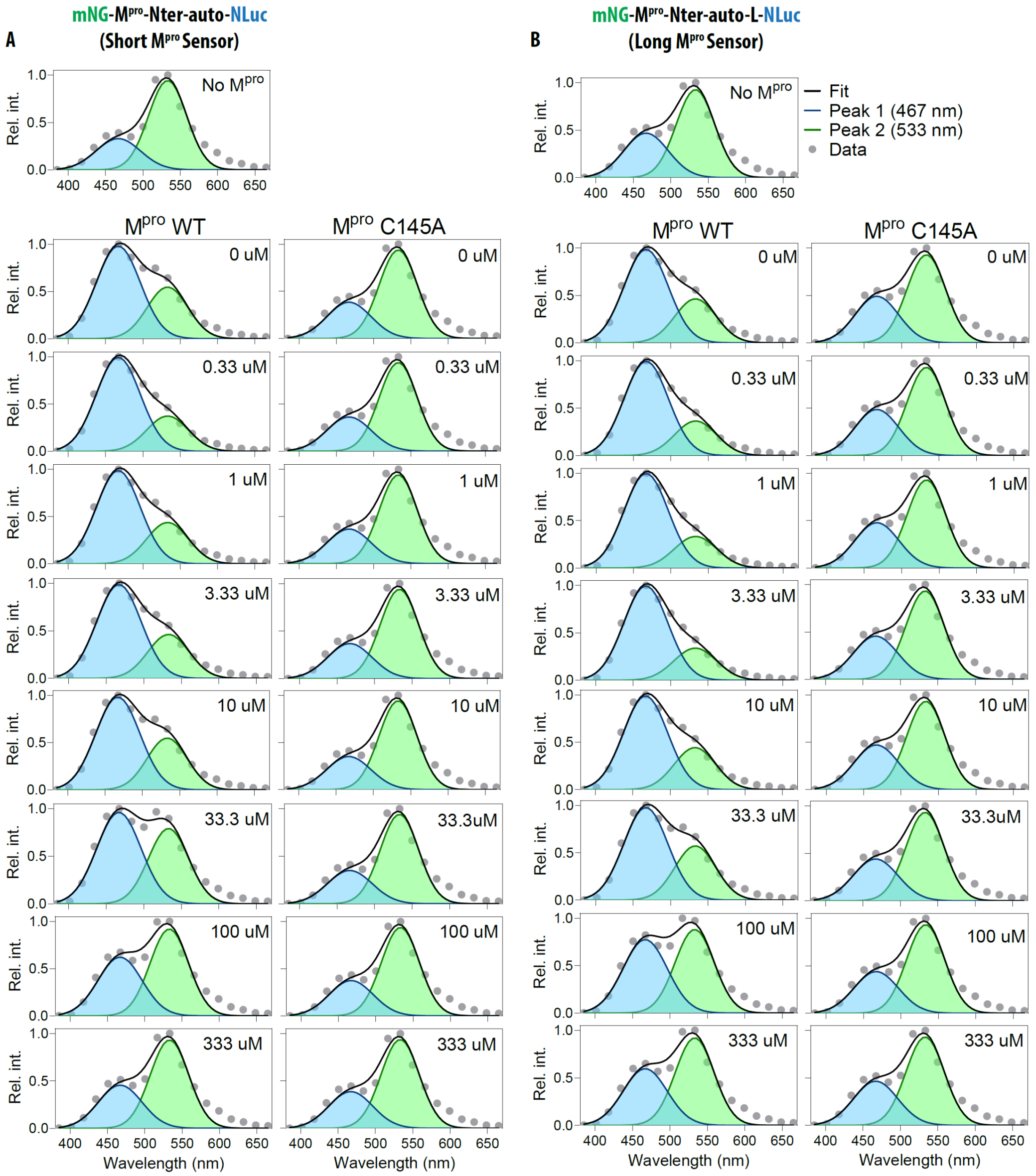

Supplementary Figure 8. GC376-mediated M^{pro} inhibition monitored in live cells. (A,B) Graphs showing bioluminescence spectra of the short (A) and long (B) M^{pro} sensor constructs in cells treated with the indicated concentrations of GC376 inhibitor in cells co-expressing either the WT or the C145A mutant M^{pro} protease. The bioluminescence spectra of the No M^{pro} control is also shown. Data were fit to a two Gaussian model reflecting mNG fluorescence and NLuc

bioluminescence peaks. Note the dose-dependent inhibition of protease activity of WT M^{pro} in cleaving both the short (A) as well as the long (B) sensors which is evident from the increased intensity of mNG peak (533 nm) of the sensor constructs. Data shown are mean ± S.D. from a representative of independent experiments, with each experiment performed in triplicates.

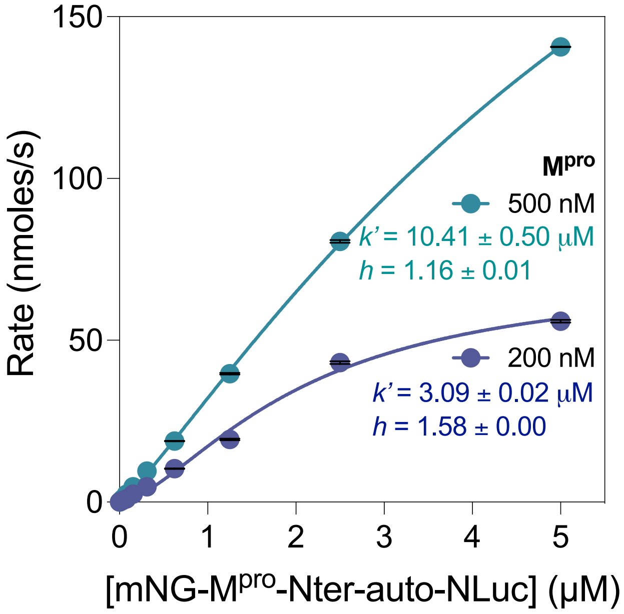

Supplementary Figure 9. In vitro enzyme kinetics assay using the short M^{pro} sensor. Graphs showing kinetic measurements of the short M^{pro} sensor cleavage in reactions containing the indicated concentrations of M^{pro}. Data plotted are average of four measurements ± SD and fit to the allosteric sigmoidal equation in GraphPad Prism. Note the decrease in the Hill coefficient (h) at 500 nM of M^{pro}.

Supplementary References

1. Gordon, D.E. et al. A SARS-CoV-2 protein interaction map reveals targets for drug repurposing. *Nature* (2020).
2. den Hamer, A. et al. Bright Bioluminescent BRET Sensor Proteins for Measuring Intracellular Caspase Activity. *ACS Sens* **2**, 729-734 (2017).
3. Bradford, M.M. A rapid and sensitive method for the quantitation of microgram quantities of protein utilizing the principle of protein-dye binding. *Anal Biochem* **72**, 248-54 (1976).
