# Supplementary material for: A genetically encoded BRET-based SARS-CoV-2 Mpro protease activity sensor
Source: Commun Chem. 2022 Sep 28;5:117. doi: 10.1038/s42004-022-00731-2 (PMC9516532; doi:10.1038/s42004-022-00731-2)
Supplement: Supplementary file 3 — Description of Additional Supplementary Files [file 42004_2022_731_MOESM3_ESM.docx]

Description of Additional Supplementary Files

**File name:** Supplementary Movie 1

**Description:** Movie showing 1 μs GaMD simulation of the short peptide.

**File name:** Supplementary Movie 2

**Description:** Movie showing 1 μs GaMD simulation of the long peptide.
