# Supplementary material for: A genetically encoded BRET-based SARS-CoV-2 Mpro protease activity sensor
Source: Commun Chem. 2022 Sep 28;5:117. doi: 10.1038/s42004-022-00731-2 (PMC9516532; doi:10.1038/s42004-022-00731-2)
Supplement: Supplementary file 6 — Reporting Summary [file 42004_2022_731_MOESM6_ESM.pdf]

Corresponding author(s): Kabir H Biswas

Last updated by author(s): May 30, 2022

Reporting Summary

Nature Research wishes to improve the reproducibility of the work that we publish. This form provides structure for consistency and transparency in reporting. For further information on Nature Research policies, see our Editorial Policies and the Editorial Policy Checklist.

Statistics

For all statistical analyses, confirm that the following items are present in the figure legend, table legend, main text, or Methods section.

n/a	Confirmed	
☐	☒	The exact sample size (*n*) for each experimental group/condition, given as a discrete number and unit of measurement
☐	☒	A statement on whether measurements were taken from distinct samples or whether the same sample was measured repeatedly
☐	☒	The statistical test(s) used AND whether they are one- or two-sided<br>*Only common tests should be described solely by name; describe more complex techniques in the Methods section.*
☒	☐	A description of all covariates tested
☒	☐	A description of any assumptions or corrections, such as tests of normality and adjustment for multiple comparisons
☒	☐	A full description of the statistical parameters including central tendency (e.g. means) or other basic estimates (e.g. regression coefficient) AND variation (e.g. standard deviation) or associated estimates of uncertainty (e.g. confidence intervals)
☒	☐	For null hypothesis testing, the test statistic (e.g. *F*, *t*, *r*) with confidence intervals, effect sizes, degrees of freedom and *P* value noted<br>*Give P values as exact values whenever suitable.*
☒	☐	For Bayesian analysis, information on the choice of priors and Markov chain Monte Carlo settings
☒	☐	For hierarchical and complex designs, identification of the appropriate level for tests and full reporting of outcomes
☒	☐	Estimates of effect sizes (e.g. Cohen's *d*, Pearson's *r*), indicating how they were calculated

Our web collection on statistics for biologists contains articles on many of the points above.

Software and code

Policy information about availability of computer code

Data collection	All bioluminescence and fluorescence data were collected using a TECAN Spark microplate reader that is supplied with proprietary software for instrument control and measurement specifications.
Data analysis	We have written a script for analyzing FlipGFP-based Mpro sensor fluorescence microscopy data for determining the fraction of GFP+ (Mpro cleavage-mediated activation of FlipGFP) cells and have included the script in the supplementary information document.

For manuscripts utilizing custom algorithms or software that are central to the research but not yet described in published literature, software must be made available to editors and reviewers. We strongly encourage code deposition in a community repository (e.g. GitHub). See the Nature Research guidelines for submitting code & software for further information.

Data

Policy information about availability of data

All manuscripts must include a data availability statement. This statement should provide the following information, where applicable:

- Accession codes, unique identifiers, or web links for publicly available datasets
- A list of figures that have associated raw data
- A description of any restrictions on data availability

All the relevant data of this study are available within this paper and the Supplementary Information file.

Field-specific reporting

Please select the one below that is the best fit for your research. If you are not sure, read the appropriate sections before making your selection.

☒ Life sciences ☐ Behavioural & social sciences ☐ Ecological, evolutionary & environmental sciences

For a reference copy of the document with all sections, see nature.com/documents/nr-reporting-summary-flat.pdf

Life sciences study design

All studies must disclose on these points even when the disclosure is negative.

Sample size	Live cell or in vitro assays were performed either twice or thrice (most of the experiments) in duplicates (in vitro assay data shown in figure 6) or triplicates (all other figures) and have been reported in the manuscript.
Data exclusions	N/A
Replication	Experiments reported in the manuscript have been repeated either twice or thrice.
Randomization	N/A
Blinding	N/A

Reporting for specific materials, systems and methods

We require information from authors about some types of materials, experimental systems and methods used in many studies. Here, indicate whether each material, system or method listed is relevant to your study. If you are not sure if a list item applies to your research, read the appropriate section before selecting a response.

Materials & experimental systems

n/a	Involved in the study	
☐	☒	Antibodies
☐	☒	Eukaryotic cell lines
☒	☐	Palaeontology and archaeology
☒	☐	Animals and other organisms
☒	☐	Human research participants
☒	☐	Clinical data
☒	☐	Dual use research of concern

Methods

n/a	Involved in the study	
☒	☐	ChIP-seq
☒	☐	Flow cytometry
☒	☐	MRI-based neuroimaging

Antibodies

Antibodies used	We have utilized the following commercially available antibodies in the current study:<br>1. Anti-His antibody (6x-His Tag Monoclonal Antibody (HIS.H8), Alexa Fluor 488; ThermoFisher Scientific-MA1-21315-A488; 1:5000 dilution);<br>2. Anti-Strep-tag mouse monoclonal antibody (anti-Strep-tag mouse monoclonal, C23.21; PROGEN- 910STR; 1:5000 dilution);<br>3. Secondary anti-mouse IgG HRP (Anti-Mouse Ig:HRP Donkey pAb; ECM biosciences- MS3001; 1:10000 dilution)
Validation	Validation/details for the respective antibodies are available in the indicated weblinks:<br>1. Anti-His antibody - https://www.thermofisher.com/antibody/product/6x-His-Tag-Antibody-clone-HIS-H8-Monoclonal/MA1-21315-A488<br>2. Anti-Strep-tag antibody - https://www.progen.com//detail/243b949ed0d240b589b7d027721e5515<br>3. Secondary anti-mouse IgG HRP - https://ecmbio.com/products/ms3001

Eukaryotic cell lines

Policy information about cell lines

Cell line source(s)	HEK293T
Authentication	HEK293T is very commonly used cell line and we have not authenticated the cell line other than visual inspection of the cell morphology.
Mycoplasma contamination	Not tested

Commonly misidentified lines
(See ICLAC register)

N/A
